# Effectiveness of BNT162b2 Against Infection, Symptomatic Infection, and Hospitalization Among Older Adults Aged ≥65 Years During the Delta Variant Predominance in Japan: The VENUS Study

**DOI:** 10.2188/jea.JE20230106

**Published:** 2024-06-05

**Authors:** Wataru Mimura, Chieko Ishiguro, Junko Terada-Hirashima, Nobuaki Matsunaga, Shuntaro Sato, Yurika Kawazoe, Megumi Maeda, Fumiko Murata, Haruhisa Fukuda

**Affiliations:** 1 Section of Clinical Epidemiology, Department of Data Science, Center for Clinical Sciences, National Center for Global Health and Medicine, Tokyo, Japan; 2 Department of Respiratory Medicine, Center Hospital of the National Center for Global Health and Medicine, Tokyo, Japan; 3 AMR Clinical Reference Center, National Center for Global Health and Medicine, Tokyo, Japan; 4 Clinical Research Center, Nagasaki University Hospital, Nagasaki, Japan; 5 Department of Health Care Administration and Management, Kyushu University Graduate School of Medical Sciences, Fukuoka, Japan

**Keywords:** SARS-CoV-2 infection, Delta variant, mRNA vaccine, Japan, cohort study

## Abstract

**Background:**

We evaluated the effectiveness of the BNT162b2 vaccine against infection, symptomatic infection, and hospitalization in older people during the Delta-predominant period (July 1 to September 30, 2021).

**Methods:**

We performed a population-based cohort study in an older adult population aged ≥65 years using data from the Vaccine Effectiveness, Networking, and Universal Safety Study conducted from January 1, 2019, to September 30, 2021, in Japan. We matched BNT162b2-vaccinated and -unvaccinated individuals in a 1:1 ratio on the date of vaccination of the vaccinated individual. We evaluated the effectiveness of the vaccine against infection, symptomatic infection, and coronavirus disease (COVID-19)-related hospitalization by comparing the vaccinated and unvaccinated groups. We estimated the risk ratio and risk difference using the Kaplan–Meier method with inverse probability weighting. The vaccine effectiveness was calculated as (1 − risk ratio) × 100%.

**Results:**

The study included 203,574 matched pairs aged ≥65 years. At 7 days after the second dose, the vaccine effectiveness of BNT162b2 against infection, symptomatic infection, and hospitalization was 78.1% (95% confidence interval [CI], 65.2–87.8%), 79.1% (95% CI, 64.6–88.9%), and 93.5% (95% CI, 83.7–100%), respectively.

**Conclusion:**

BNT162b2 was highly effective against infection, symptomatic infection, and hospitalization in Japan’s older adult population aged ≥65 years during the Delta-predominant period.

## INTRODUCTION

Severe acute respiratory syndrome coronavirus 2 (SARS-CoV-2) has caused the coronavirus disease (COVID-19) pandemic, which has persisted for almost 3 years since the first cases were reported at the end of 2019.^1^ Japan experienced five waves of SARS-CoV-2 infection in 2020 and 2021.^2^ The Alpha variant (B.1.1.7) circulated in May 2021 and was gradually replaced by the Delta (B. 1.617.2) variant in June 2021.^3^^,^^4^ The Delta variant reached approximately ≥80% of all Japanese regions by August 2021 and caused the fifth wave, which peaked at approximately 128 per 100,000 population in a week.^5^^,^^6^ The circulation of the Delta variant lasted until the Omicron variant (B.1.1.529) surged. The Delta variant showed higher transmissibility and virulence than the Alpha variant.^7^^,^^8^ Consequently, COVID-19 cases, hospitalizations, and severe cases were higher than in the previous four waves.^2^

mRNA vaccines (BNT162b2 and mRNA-1273) against SARS-CoV-2 were approved in Japan on February 14, 2021, and May 21, 2021, respectively.^9^ mRNA vaccines have shown high efficacy and effectiveness in clinical trials and observational studies.^10^^–^^12^ A clinical trial of the BNT162b2 primary series showed 94.8% (95% confidence interval [CI], 89.8–97.6%) efficacy against COVID-19 in persons aged ≥16 years, and an observational study found a similar level of effectiveness. Several case-control studies have confirmed the effectiveness of mRNA vaccines against symptomatic infections in Japan.^13^^–^^15^ However, the report of vaccine effectiveness against severe COVID-19 in the older adult population during the Delta-predominant period has been limited, despite the high proportion of a population aged ≥65 years in Japan (28.8% in 2020).^16^ Older adults have an increased risk of severe COVID-19 compared with individuals aged 18–39 years.^17^ Compared with those aged 18–39 years, the relative risk of intensive care unit admission has been reported as 1.43 in those aged 65–74 years, and 1.35 in those aged ≥75 years. Compared with those aged 18–39 years, the relative risk of death has been reported as 6.14 in those aged 65–74 years, and 8.66 in those aged ≥75 years. Other risk factors for severe COVID-19 include underlying diseases, such as cancer, chronic kidney disease, chronic lung diseases, and dementia.^18^^,^^19^ Therefore, considering many background factors, it is necessary to assess the effectiveness of the vaccine against severe COVID-19 in older adults.

We developed a database to assess vaccine effectiveness and safety and linked routine vaccination records and many data sources in the Vaccine Effectiveness, Networking, and Universal Safety (VENUS) Study.^20^ We have previously reported the results of the VENUS Study, estimating the vaccine effectiveness of mRNA vaccines against infection (83.8%) and symptomatic infection (89.8%) at 7 days after the second dose in the population aged 16–64 years during the Delta-predominant period using COVID-19 case data linked with COVID-19 vaccination data.^21^ However, there was no information on the severity of COVID-19 or comorbidities. Therefore, we used VENUS Study data linked with medical claims to adjust background factors and assess the effectiveness against COVID-19-related hospitalization, in addition to infection and symptomatic infection. Moreover, for future development of healthcare and surveillance of infectious disease, it is important to show that vaccine effectiveness can be estimated in a real-world setting based on the secondary use of administrative data routinely collected for other purposes. In this study, we aimed to evaluate the vaccine effectiveness of BNT162b2 against infection, symptomatic infection, and hospitalization among older adults aged ≥65 years during the Delta-predominant period in Japan.

## METHODS

### Data source

We used municipality-based data from January 1, 2019, to September 30, 2021, from the VENUS Study in Japan. The data were from four municipalities. Two municipalities are located in the Kanto region, one in the Chugoku region, and one in the Chubu region. The Kanto region is an eastern area that includes Tokyo. The Chugoku region is the western area of Honshu, the largest island in Japan. The Chubu region is in the middle of Honshu. The data included the Health Center Real-time information-sharing System on COVID-19 (HER-SYS), Vaccination Record System (VRS), and medical claims.^22^^,^^23^ The HER-SYS contains information on SARS-CoV-2 testing (type of specimens collected, collection date, type of testing, and results). The VRS includes the vaccination date and the products’ names (first, second, and third doses). The medical claims contain data on residents enrolled in the National Health Insurance System and the Latter-Stage Older Persons Health Care System.^20^ The claims data include residents’ demographics (age and sex), medications, procedures, and surgeries. The record linkage of the HER-SYS, VRS, and claims was performed individually in each municipality. All data provided by the four municipalities followed the ordinance for the protection of personal information and the related regulations. All data were anonymized, and the Kyushu University Institutional Review Board for Clinical Research approved the study (No. 2021-399). In addition, the requirement for informed consent was waived based on the Japanese ethical guidelines, as this secondary analysis used routinely collected anonymized data by the municipalities.

### Study design and setting

This population-based cohort study included individuals aged ≥65 years in April 2021. The vaccination campaign for the general population, prioritizing older adults aged ≥65 years, was initiated on April 12, 2021. As a non-pharmaceutical intervention, states of emergency or semi-emergency were declared in each prefecture considering the local viral circulation, and preventive behavior such as wearing masks, social distancing, handwashing, and ventilation were required throughout the study period. Vaccination uptake among older adults was high after the campaign began, and vaccination coverage reached 79.1% by July 30, 2021.^24^ Subsequently, the coverage of the primary series reached approximately 90% in October 2021 and 91.1% by the end of December 2021. BNT162b2 was the main vaccine used for the primary vaccination of older adults.^25^ Therefore, we identified the period in which each individual was unvaccinated and vaccinated with BNT162b2 to evaluate the primary series of vaccinations by BNT162b2. Individuals vaccinated with BNT162b2 were included from the date of the first dose, defined as the cohort entry date. We excluded the following individuals from the study: (1) individuals vaccinated before April 12, 2021; (2) individuals whose most recent claims records were before their cohort entry date (to exclude those who withdrew from their insurance plan, died, or moved to another municipality); (3) individuals without any claim records in the year before the cohort entry date, and (4) individuals with previous COVID-19 before the cohort entry date. To compare the vaccinated and unvaccinated groups, the unvaccinated group was selected by random sampling of unvaccinated individuals on each vaccinated individual’s cohort entry date. Thus, the cohort entry date of each unvaccinated individual was defined by the same calendar date as the cohort entry date of the paired vaccinated individual. The exclusion criteria for the unvaccinated group were the same as those for the vaccinated group. The matched individuals were followed up from the day of each vaccination and ended on the first day that any of the following criteria were met: (1) the day of outcome occurrence; (2) the day before vaccination among individuals included in the unvaccinated group; (3) the end of the assessment of vaccination status; (4) administration of another product (mRNA-1273 or ChAdOx1 nCoV-19); (5) the last claims record during the study period; (6) administration of an early second dose (<19 days after the first dose); and (7) the end of the study period (September 30, 2021). If an individual in the unvaccinated group was vaccinated, the pair was censored on the same calendar date. Then the newly vaccinated individual became the matching candidate to enter the vaccinated group.

We categorized vaccination status into five categories: unvaccinated, 0–13 days after the first dose, 14 days after the first dose until 20 days or the second dose, 21 days after the first dose until 7 days or 0–6 days after the second dose, and 7 days or more after the second dose. In addition, to assess the vaccine effectiveness against the Delta variant, we used the period between July 1, 2021, and September 30, 2021, according to the previous report; and we defined the period as the Delta-predominant period.

### Outcomes

We defined three outcomes (infection, symptomatic infection, and COVID-19-related hospitalization). Infection was defined as a test positive for SARS-CoV-2 using a nucleic acid amplification test or an antigen test (symptomatic and asymptomatic infections). Symptomatic infection was defined as a test positive for SARS-CoV-2, with COVID-19-related symptoms (eg, fever, cough, pneumonia, fatigue, headache). A combination of the HER-SYS and claims data identified COVID-19-related hospitalization. COVID-19-related hospitalization was defined as hospitalization between 2 days before and 14 days after the SARS-CoV2-positive specimen was collected.

### Covariates

We obtained information on several covariates to account for the risk of developing severe COVID-19. Participants’ age and sex were assessed at cohort entry. We used International Statistical Classification of Diseases and Related Health Problems, 10^th^ revision (ICD-10) codes to identify each participant’s underlying disease during the year before the cohort entry date. These underlying diseases included chronic pulmonary disease, cardiovascular disease, cerebrovascular disease, rheumatic disease, diabetes, dementia, renal disease, liver disease, dementia, cancer, dyslipidemia, and hypertension (eTable 1). Dyslipidemia and hypertension were defined as the combination of the diagnosis and medication more than two times or more during the year before the cohort entry date. Furthermore, we calculated each participant’s number of clinic/hospital visits and COVID-19 testing in the year before their cohort entry date as an indicator of healthcare-seeking behavior.

### Statistical analysis

We summarized individual characteristics as the mean (standard deviation [SD]), median (interquartile range [IQR]), or number (%). The baseline covariates of the vaccinated and unvaccinated groups were compared using standardized differences. We considered that a standardized difference <0.1 indicated a negligible imbalance. To assess vaccine effectiveness during the Delta-predominant period, we created four analysis groups by identifying the matched pairs still at risk of SARS-CoV-2 infection at the start of each vaccination status (0–13 days after the first dose, 14 days after the first dose until 20 days or the second dose, 21 days after the first dose until 7 days or 0–6 days after the second dose, and 7 days after the second dose). In each analysis group, we estimated the propensity score using logistic regression, including the covariates (age as a continuous variable, sex, municipality, each underlying disease, the number of clinical/hospital visits during the year before cohort entry as a continuous variable, and the number of SARS-CoV-2 tests conducted during the year before cohort entry as a continuous variable). The Kaplan–Meier estimator was used to estimate the cumulative incidence of outcomes in the matched cohort and analysis groups. Adjustment of confounding factors for risk ratios was performed by inverse probability weighting using the propensity score. The risk ratios were calculated and compared between the vaccinated and unvaccinated groups using the adjusted Kaplan–Meier estimator. Vaccine effectiveness was calculated as (1 − risk ratio) × 100%. The risk difference was calculated as the number of events per 10,000 persons in the unvaccinated minus that in the vaccinated group. The 95% confidence interval (CI) was calculated using percentile bootstrap methods with 1,000 repetitions. For sensitivity analysis, we changed the Delta-predominant period to August 1, 2021 to September 30, 2021, because the Alpha variant was cocirculating in July 2021. In addition, we performed Cox regression analysis with inverse probability weighting accounting for the matched pairs as the cluster. The vaccine effectiveness was calculated as (1 − hazard ratio) × 100%. All statistical analyses were performed using R statistical software version 4.1.2 (R Foundation for Statistical Computing, Vienna, Austria).

## RESULTS

**Figure 1.**
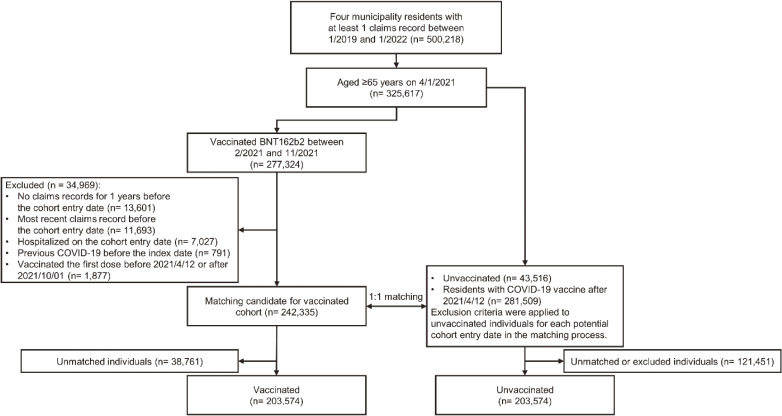
Flow chart. COVID-19, coronavirus disease 2019.

During the study period, 325,291 residents aged ≥65 years were identified in the four municipalities (Figure 1). Among these residents, 242,335 vaccinated and 325,025 unvaccinated individuals were identified as matching candidates in this study. In matching the residents in a 1:1 ratio of vaccinated to unvaccinated individuals, 203,574 matched pairs were included in the study. In the vaccinated group, 42,311 (20.8%), 123,058 (60.9%), and 28,511 (14.0%) patients were administered a first dose in May, June, and July 2022, respectively. In addition, 202,797 (99.6%) were vaccinated with a second dose, and 193,463 (95.4%) were vaccinated with a second dose on 21 days after the first dose. In the unvaccinated group, 187,881 individuals (92.2%) were vaccinated after cohort entry. The baseline characteristics of the matched vaccinated and unvaccinated groups are presented Table 1 and eTable 2. The mean age was 78.2 (SD, 7.2) years, and 120,053 (59.0%) were women in the vaccinated group. In the unvaccinated group, the mean age was 77.4 (SD, 7.3) years, and 118,249 (58.1%) were women. The age of the vaccinated group was higher than that of the unvaccinated group, with a standardized difference of 0.119. The standardized differences in comorbidities were <0.1; thus, there were no important differences between the vaccinated and unvaccinated cohorts.

**Table 1.** Baseline characteristics in the matched cohort

	Vaccinated, *N* = 203,574	Unvaccinated, *N* = 203,574	Standardized difference
Age, years, mean (SD)	78.2 (7.2)	77.4 (7.3)	0.119
Age groups, years, *n* (%)			0.128
65–74	70,848 (34.8%)	83,316 (40.9%)	
75–84	91,384 (44.9%)	83,877 (41.2%)	
85–94	37,773 (18.6%)	33,430 (16.4%)	
≥95	3,569 (1.8%)	2,951 (1.4%)	
Sex, *n* (%)			0.018
Men	83,521 (41.0%)	85,325 (41.9%)	
Women	120,053 (59.0%)	118,249 (58.1%)	
Cancer, *n* (%)	31,737 (15.6%)	31,971 (15.7%)	0.003
Chronical pulmonary disease, *n* (%)	40,345 (19.8%)	38,442 (18.9%)	0.024
Cardiovascular disease, *n* (%)	68,475 (33.6%)	65,017 (31.9%)	0.036
Cerebrovascular disease, *n* (%)	43,185 (21.2%)	41,004 (20.1%)	0.026
Renal disease, *n* (%)	12,174 (6.0%)	11,719 (5.8%)	0.010
Liver disease, *n* (%)	43,415 (21.3%)	43,181 (21.2%)	0.003
Diabetes, *n* (%)	20,779 (10.2%)	21,063 (10.3%)	0.005
Dementia, *n* (%)	18,479 (9.1%)	14,978 (7.4%)	0.063
Rheumatic disease, *n* (%)	8,964 (4.4%)	8,812 (4.3%)	0.004
Hypertension, *n* (%)	121,204 (59.5%)	117,718 (57.8%)	0.042
Dyslipidemia, *n* (%)	82,630 (40.6%)	79,783 (39.2%)	0.030
Number of clinic/hospital visits in the past year, Median (IQR)	18.0 (11.0, 30.0)	16.0 (10.0, 28.0)	0.071
Number of previous COVID-19 testing, *n* (%)			0.030
0	190,890 (93.8%)	189,623 (93.1%)	
1	10,047 (4.9%)	10,700 (5.3%)	
≥2	2,637 (1.3%)	3,251 (1.6%)	
City, *n* (%)			0.171
A	44,207 (21.7%)	43,440 (21.3%)	
B	34,920 (17.2%)	47,502 (23.3%)	
C	52,134 (25.6%)	51,962 (25.5%)	
D	72,313 (35.5%)	60,670 (29.8%)	

COVID-19, coronavirus disease; IQR, interquartile range; SD, standard deviation.

**Figure 2.**
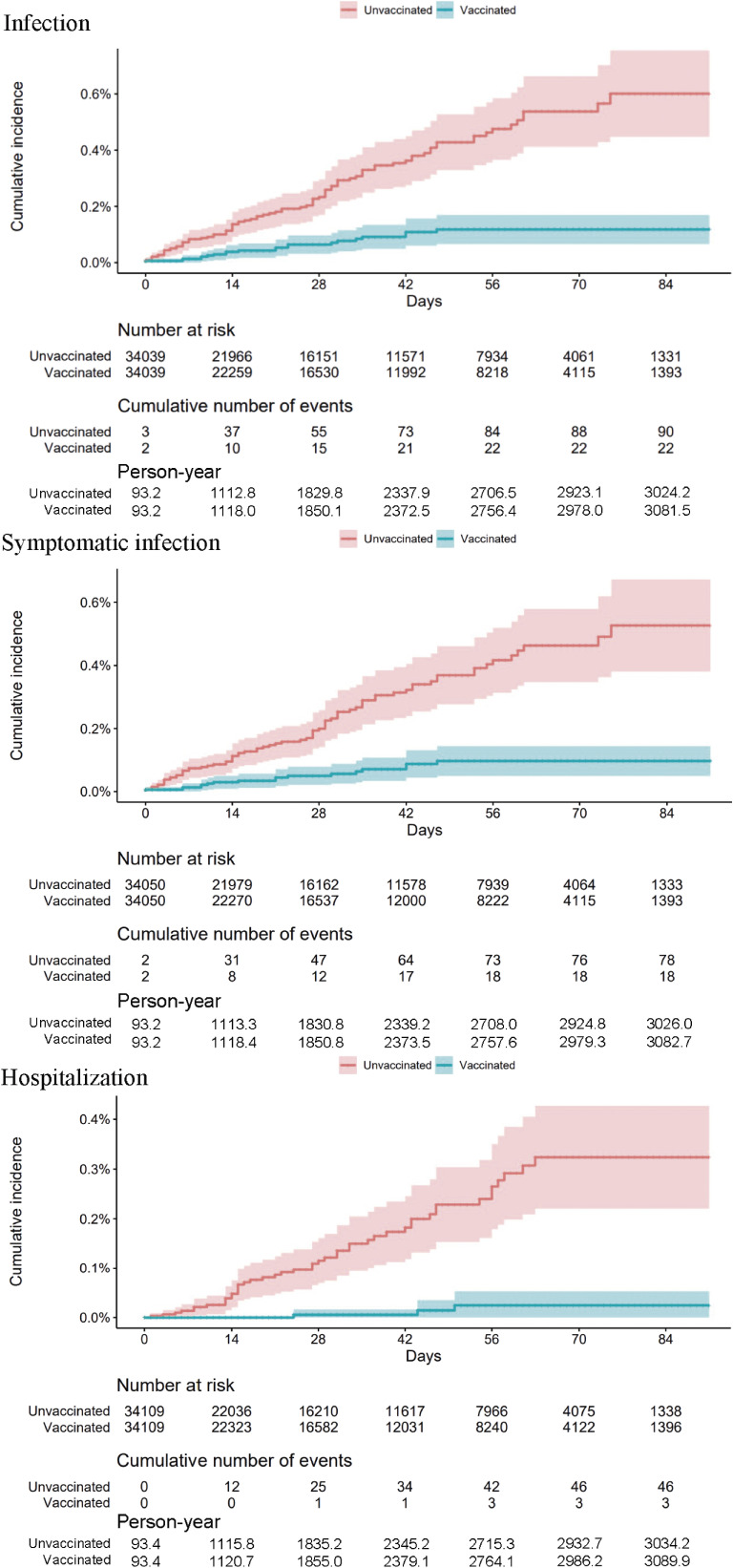
Cumulative incidence of infection, symptomatic infection, and hospitalization after restricting the population at 7 days after the second dose

The number of infections from April 12, 2021, to September 30, 2021, was 82 and 184 in the matched vaccinated and unvaccinated groups, respectively. The cumulative incidence in the matched cohort is shown in eFigure 1. The analysis group for each vaccination status (0–13 days after the first dose, 14 days after the first dose until 20 days or second dose, 21 days after the first dose until 7 days or 0–6 days after the second dose, and 7 days after the second dose) included 58,185, 42,632, 37,477, and 34,039 individuals, respectively. The standardized difference of the covariates was <0.1 in all analysis groups. The median follow-up period was 26 (IQR, 8–54) days in the analysis group to assess effectiveness 7 days after the second dose. The cumulative incidence of infection, symptomatic infection, and hospitalization in the analysis groups that restricted the assessment of vaccine effectiveness are shown in Figure 2 (effectiveness 7 days after the second dose), eFigure 2, eFigure 3, and eFigure 4 (0–13 days after the first dose, 14 days after the first dose until 20 days or second dose, 21 days after the first dose until 7 days or 0–6 days after the second dose). The BNT162b2 vaccine was effective against infection, symptomatic infection, and hospitalization among older adults aged ≥65 years for each vaccination status (Table 2). Seven days after the second dose, the vaccine effectiveness was 78.1% (95% CI, 65.2–87.8%) against infection, 79.1% (95% CI, 64.6–88.9%) against symptomatic infection, and 93.5% (95% CI, 83.7–100%) against hospitalization. The results of the sensitivity analyses were consistent with the main results (eTable 3 and eTable 4).

**Table 2.** Effectiveness of BNT162b2 vaccine in the older adults aged ≥65 years during the Delta-predominant period

	Number at risk at the start of follow-up	Events	Events/10,000 persons (95% CI)^a^	Risk difference (95% CI)^a,b^	Vaccine effectiveness (95% CI)^a^
**Infection**					
Unvaccinated	58,185	26	4.6 (2.7–6.4)	Ref	Ref
0–13 days after first dose	58,185	25	4.7 (2.9–6.6)	−0.1 (−2.8 to 2.5)	12.9 (−67.5 to 53.7)
Unvaccinated	42,632	16	4.7 (2.4–7.4)	Ref	Ref
14–20 days after first dose or the day before second dose 13 days after the second dose	42,632	12	3.6 (1.8–5.9)	1.2 (−2.2 to 4.5)	26.2 (−74.0 to 69.0)
Unvaccinated	37,477	10	3.4 (1.4–5.6)	Ref	Ref
21–27 days after first dose or 0–6 days after the second dose	37,477	11	4.1 (1.7–7.0)	−0.7 (−4.2 to 2.7)	−7.4 (−190.0 to 62.2)
Unvaccinated	34,039	90	26.5 (21.2–32.4)	Ref	Ref
7 days or more after the second dose	34,039	22	7.6 (4.4–11.4)	18.9 (12.2–25.5)	78.1 (65.2–87.8)
**Symptomatic infection**					
Unvaccinated	58,185	23	4.1 (2.3–5.9)	Ref	Ref
0–13 days after first dose	58,185	23	4.3 (2.5–6.1)	−0.3 (−2.7 to 2.2)	8.0 (−81.8 to 51.4)
Unvaccinated	42,635	14	4.3 (2.2–6.7)	Ref	Ref
14–20 days after first dose or the day before second dose 13 days after the second dose	42,635	9	2.6 (1.0–4.6)	1.7 (−1.6 to 4.8)	40.3 (−57.0 to 80.6)
Unvaccinated	37,485	8	2.7 (0.9–4.8)	Ref	Ref
21–27 days after first dose or 0–6 days after the second dose	37,485	9	3.5 (1.4–6.2)	−0.8 (−4.0 to 2.2)	−15.4 (−285.6 to 61.9)
Unvaccinated	34,050	78	22.8 (18.0–27.9)	Ref	Ref
7 days or more after the second dose	34,050	18	6.4 (3.5–9.6)	16.4 (10.3–22.5)	79.1 (64.6–88.9)
**Hospitalization**					
Unvaccinated	58,186	4	0.7 (0.1–1.4)	Ref	Ref
0–13 days after first dose	58,186	1	0.3 (0.0–1.0)	0.3 (−0.7 to 1.3)	76.0 (−93.3 to 100.0)
Unvaccinated	42,670	2	0.8 (0.0–2.1)	Ref	Ref
14–20 days after first dose or the day before second dose 13 days after the second dose	42,670	2	0.5 (0.0–1.3)	0.3 (−1.0 to 1.6)	26.6 (−391.8 to 100.0)
Unvaccinated	37,540	3	1.1 (0.0–2.7)	Ref	Ref
21–27 days after first dose or 0–6 days after the second dose	37,540	2	0.5 (0.0–1.3)	0.6 (−0.7 to 2.1)	55.9 (−176.1 to 100.0)
Unvaccinated	34,110	46	14.2 (10.1–18.5)	Ref	Ref
7 days or more after the second dose	34,110	3	0.8 (0.0–1.9)	13.4 (9.3–17.9)	93.5 (83.7–100.0)

CI, confidence interval.

^a^Adjusted by inverse probability weighting using the propensity score.

^b^The risk differences were calculated as unvaccinated minus vaccinated groups.

## DISCUSSION

This study assessed the effectiveness of BNT162b2 against infections, symptomatic infections, and hospitalization during the Delta-predominant period among older adults in Japan. Our results showed that the effectiveness of BNT162b at 7 days after the second dose was 78.1%, 79.1%, and 93.5% against infection, symptomatic infection, and hospitalization, respectively, in the population aged ≥65 years.

A previous study with a test-negative design in Japan showed that the effectiveness of the BNT162b2 vaccine against symptomatic infection was 85.8% in the population aged ≥65 years during the Delta variant epidemic.^13^ In Italy, the effectiveness of the mRNA vaccines (BNT162b2 or mRNA-1273) against infection was 56.4% in the 60–79 years age group and 35.0% in those aged ≥80 years. Furthermore, its effectiveness against severe COVID-19 was 90.5% in those aged 60–79 years and 76.7% in those aged ≥80 years.^26^ In the United States, the effectiveness of BNT162b2 was 79.6%, 98.0%, and 95.1% against symptomatic infection, hospitalization, and death, respectively, in patients aged ≥65 years.^27^ In our study, the vaccine effectiveness 7 days after the second dose was comparable to that in previous studies, which found that its effectiveness in older adults and against hospitalization was higher than its effectiveness against other outcomes.

The timing of the vaccination program would contribute to the control and reduction of COVID-19. The initiation of the vaccination program for the general population in Japan started on April 14, 2021; however, it had been delayed compared with other countries, such as Israel, the United Kingdom, and the United States. After the vaccination program began in Japan, vaccination coverage of the primary dose rapidly increased, especially in the older population aged ≥65 years. Due to performing fast vaccination, the number of COVID-19 cases, severe COVID-19 cases, and clusters in long-term care facilities decreased among older adults, although the Delta wave started in July 2021. In this study, the calculated number needed to vaccinate (NNV), based on the difference in the Kaplan–Meier estimated cumulative incidence at the end of the follow-up period, was 331.5. However, the NNV varies depending on the setting. For example, one study found that the NNV to prevent one hospitalization was 46–100 during the Omicron period in the United States.^28^ Moreover, a study estimated a large reduction in COVID-19 cases and deaths attributable to the vaccination program during the Delta wave, especially in the older population.^29^ In the United States, a substantial reduction across whole generations was estimated.^30^ Those differences would be affected by the timing of the vaccination program or measurement in each country. Therefore, it is important to assess the impact of the vaccination program to discuss future measures against infectious diseases. We provide useful data to assess the impact because the effectiveness from infection to hospitalization was consistent in the study population.

Our previous reports on the VENUS Study showed COVID-19 vaccine effectiveness against infection and symptomatic infection among the population aged <65 years during the waves of the Delta variant.^21^ We did not consider the underlying diseases because the study used only the HER-SYS and VRS data. These data did not include sufficient information on the risk factors related to underlying diseases for severe COVID-19. Older age is one of the risk factors for severe COVID-19; moreover, these individuals are more likely to have underlying diseases. Therefore, it was necessary to consider underlying diseases to assess the effectiveness of COVID-19-related hospitalization among older adults in this study. To the best of our knowledge, this study is the first to use HER-SYS, VRS, and claims data, although several studies have assessed the COVID-19 vaccine effectiveness in Japan.^13^^–^^15^^,^^21^^,^^31^^,^^32^

This study illustrates the value of linking routine claims data from the Japanese healthcare insurance system to data collected for monitoring infectious diseases and vaccination. This study has several limitations. First, we could not identify the variant type because we could not obtain information about the genome sequence. We restricted the analysis to the Delta-predominant period (July 1, 2021 to September 30, 2021); however, the Alpha variant was still circulating during this period, especially in July 2021. The Omicron variant would not be included so that the variant circulated mainly from January 2022. Second, we could not assess vaccine effectiveness at the municipality level owing to sample size. The incidence of COVID-19 was lower in Japan than in other countries until the Omicron variant circulated.^33^ Moreover, after starting vaccination in each municipality, almost all residents were rapidly vaccinated, especially the older adults (aged ≥65 years), who were prioritized to be vaccinated over the younger population. Therefore, unvaccinated residents in our study population became vaccinated very soon after their cohort entry, and the pairs matched with vaccinated and unvaccinated residents were censored in pairs. Third, there may be residual confounding in this study. For example, some factors related to severe outcomes, such as obesity or smoking status, were not identified. In addition, owing to a lack of information, we were unable to adequately consider health-seeking behavior. Fifth, there may be some selection bias because this cohort comprised individuals with at least one claim record before and after the cohort entry date. Thus, healthy older individuals aged ≥65 years who rarely see a doctor and uninfected individuals were not included in the study. This bias would have led to underestimation of the vaccine effectiveness in this study. Sixth, this study assessed underlying disease based on claims data; thus, there could be some misclassification of the underlying diseases. However, we assessed underlying diseases, including risk factors for severe COVID-19, and the covariates related to health-care-seeking behavior using claims data from the VENUS Study.

### Conclusion

This large municipality-based cohort study from the VENUS Study showed the effectiveness of the BNT162b2 vaccine among older adults aged ≥65 years during the Delta-predominant period in four municipalities in Japan. Its effectiveness against infection, symptomatic infection, and hospitalization was high.

## SUPPLEMENTARY MATERIAL

eTable 1. Covariates definitions

eTable 2. Baseline characteristics in the matched cohort stratified by municipality

eTable 3. Effectiveness of BNT162b2 vaccine in the older adults aged ≥65 years during the Delta-predominant period (August 1, 2021–September 30, 2021)

eTable 4. BNT162b2 vaccine effectiveness using Cox proportional hazard model during the Delta-predominant period (July 1, 2021–September 30, 2021)

eFigure 1. Cumulative incidence of SARS-CoV-2 infection in the matched cohort between April 12, 2021 and September 30, 2021

eFigure 2. Cumulative incidence of SARS-CoV-2 infection after restricting the population still at risk at the start each period. (**A**) 0–13 days after the first dose, (**B**) 14–20 days after the first dose or the day before the second dose or 14 days after the second dose, (**C**) 21–27 days after the first dose or 0–6 days after the second dose

eFigure 3. Cumulative incidence of symptomatic SARS-CoV-2 infection after restricting the population still at risk at the start each period. (**A**) 0–13 days after the first dose, (**B**) 14–20 days after the first dose or the day before the second dose or 14 days after the second dose, (**C**) 21–27 days after the first dose or 0–6 days after the second dose

eFigure 4. Cumulative incidence of hospitalization related to SARS-CoV-2 infection in the population still at risk at the start each period. (**A**) 0–13 days after the first dose, (**B**) 14–20 days after the first dose or the day before the second dose or 14 days after the second dose, (**C**) 21–27 days after the first dose or 0–6 days after the second dose
