# Supplementary material for: Effectiveness of BNT162b2 Against Infection, Symptomatic Infection, and Hospitalization Among Older Adults Aged ≥65 Years During the Delta Variant Predominance in Japan: The VENUS Study
Source: J Epidemiol. 2024 Jun 5;34(6):278–85. doi: 10.2188/jea.JE20230106 (PMC11078592; doi:10.2188/jea.JE20230106)
Supplement: Supplementary file 1 [file je-34-278-s001.pdf]

eTable 1. Covariates definitions

Covariate	Definition	Period
Cancer	At least one of the following diagnoses (ICD-10 codes): C00.0/C00.1/C00.2/C00.3/C00.4/C00.5/C00.6/C00.8/C00.9/C01/C02.0/C02.1/C02.2/C02.3/C02.4/C02.8/C02.9/C03.0/C03.1/C03.9/C04.0/C04.1/C04.8/C04.9/C05.0/C05.1/C05.2/C05.8/C05.9/C06.0/C06.1/C06.2/C06.8/C06.9/C07/C08.0/C08.1/C08.8/C08.9/C09.0/C09.1/C09.8/C09.9/C10.0/C10.1/C10.2/C10.3/C10.4/C10.8/C10.9/C11.0/C11.1/C11.2/C11.3/C11.8/C11.9/C12/C13.0/C13.1/C13.2/C13.8/C13.9/C14.0/C14.2/C14.8/C15.0/C15.1/C15.2/C15.3/C15.4/C15.5/C15.8/C15.9/C16.0/C16.1/C16.2/C16.3/C16.4/C16.5/C16.6/C16.8/C16.9/C17.0/C17.1/C17.2/C17.3/C17.8/C17.9/C18.0/C18.1/C18.2/C18.3/C18.4/C18.5/C18.6/C18.7/C18.8/C18.9/C19/C20/C21.0/C21.1/C21.2/C21.8/C22.0/C22.1/C22.2/C22.3/C22.4/C22.7/C22.9/C23/C24.0/C24.1/C24.8/C24.9/C25.0/C25.1/C25.2/C25.3/C25.4/C25.7/C25.8/C25.9/C26.0/C26.1/C26.8/C26.9/C30.0/C30.1/C31.0/C31.1/C31.2/C31.3/C31.8/C31.9/C32.0/C32.1/C32.2/C32.3/C32.8/C32.9/C33/C34.0/C34.1/C34.2/C34.3/C34.8/C34.9/C37/C38.0/C38.1/C38.2/C38.3/C38.4/C38.8/C39.0/C39.8/C39.9/C40.0/C40.1/C40.2/C40.3/C40.8/C40.9/C41.0/C41.1/C41.2/C41.3/C41.4/C41.8/C41.9/C43.0/C43.1/C43.2/C43.3/C43.4/C43.5/C43.6/C43.7/C43.8/C43.9/C45.0/C45.1/C45.2/C45.7/C45.9/C46.0/C46.1/C46.2/C46.3/C46.7/C46.8/C46.9/C47.0/C47.1/C47.2/C47.3/C47.4/C47.5/C47.6/C47.8/C47.9/C48.0/C48.1/C48.2/C48.8/C49.0/C49.1/C49.2/C49.3/C49.4/C49.5/C49.6/C49.8/C49.9/C50.0/C50.1/C50.2/C50.3/C50.4/C50.5/C50.6/C50.8/C50.9/C51.0/C51.1/C51.2/C51.8/C51.9/C52/C53.0/C53.1/C53.8/C53.9/C54.0/C54.1/C54.2/C54.3/C54.8/C54.9/C55/C56/C57.0/C57.1/C57.2/C57.3/C57.4/C57.7/C57.8/C57.9/C58/C60.0/C60.1/C60.2/C60.8/C60.9/C61/C62.0/C62.1/C62.9/C63.0/C63.1/C63.2/C63.7/C63.8/C63.9/C64/C65/C66/C67.0/C67.1/C67.2/C67.3/C67.4/C67.5/C67.6/C67.7/C67.8/C67.9/C68.0/C68.1/C68.8/C68.9/C69.0/C69.1/C69.2/C69.3/C69.4/C69.5/C69.6/C69.8/C69.9/C70.0/C70.1/C70.9/C71.0/C71.1/C71.2/C71.3/C71.4/C71.5/C71.6/C71.7/C71.8/C71.9/C72.0/C72.1/C72.2/C72.3/C72.4/C72.5/C72.8/C72.9/C73/C74.0/C74.1/C74.9/C75.0/C75.1/C75.2/C75.3/C75.4/C75.5/C75.8/C75.9/C76.0/C76.1/C76.2/C76.3/C76.4/C76.5/C76.7/C76.8/C77.0/C77.1/C77.2/C77.3/C77.4/C77.5/C77.8/C77.9/C78.0/C78.1/C78.2/C78.3/C78.4/C78.5/C78.6/C78.7/C78.8/C79.0/C79.1/C79.2/C79.3/C79.4/C79.5/C79.6/C79.7/C79.8/C80/C81.0/C81.1/C81.2/C81.3/C81.7/C81.9/C82.0/C82.1/C82.2/C82.7/C82.9/C83.0/C83.1/C83.2/C83.3/C83.4/C83.5/C83.6/C83.7/C83.8/C83.9/C84.0/C84.1/C84.2/C84.3/C84.4/C84.5/C85.0/C85.1/C85.7/C85.9/C88.0/C88.1/C88.2/C88.3/C88.7/C88.9/C90.0/C90.1/C90.2/C91.0/C91.1/C91.2/C91.3/C91.4/C91.5/C91.7/C91.9/C92.0/C92.1/C92.2/C92.3/C92.4/C92.5/C92.7/C92.9/C93.0/C93.1/C93.2/C93.7/C93.9/C94.0/C94.1/C94.2/C94.3/C94.4/C94.5/C94.7/C95.0/C95.1/C95.2/C95.7/C95.9/C96.0/C96.1/C96.2/C96.3/C96.7/C96.9/C97	1 year before the cohort entry date

Chronic pulmonary disease	At least one of the following diagnoses (ICD-10 codes): I27.8/I27.9/J40/J41.0/J41.1/J41.8/J42/J43.0/J43.1/J43.2/J43.8/J43.9/J44.0/J44.1/J44.8/J44.9/J45.0/J45.1/J45.8/J45.9/J46/J47/J60/J61/J62.0/J62.8/J63.0/J63.1/J63.2/J63.3/J63.4/J63.5/J63.8/J64/J65/J66.0/J66.1/J66.2/J66.8/J67.0/J67.1/J67.2/J67.3/J67.4/J67.5/J67.6/J67.7/J67.8/J67.9/J68.4/J70.1/J70.3	1 year before the cohort entry date
Cardiovascular disease	At least one of the following diagnoses (ICD-10 codes): I21.0/I21.1/I21.2/I21.3/I21.4/I21.9/I22.0/I22.1/I22.8/I22.9/I25.2<br>I09.9/I11.0/I13.0/I13.2/I25.5/I42.0/I42.5/I42.6/I42.7/I42.8/I42.9<br>/I43.0/I43.1/I43.2/I43.8/I50.0/I50.1/I50.9/P29.0<br>I44.1/I44.2/I44.3/I45.6/I45.9/I47.0/I47.1/I47.2/I47.9/I48/I49.0/I49.1/I49.2/I49.3/I49.4/I49.5/I49.8/I49.9/R00.0/R00.1/R00.8/T82.1/Z45.0/Z95.0<br>A52.0/I05.0/I05.1/I05.2/I05.8/I05.9/I06.0/I06.1/I06.2/I06.8/I06.9/I07.0/I07.1/I07.2/I07.8/I07.9/I08.0/I08.1/I08.2/I08.3/I08.8/I08.9/I09.1/I09.8/I34.0/I34.1/I34.2/I34.8/I34.9/I35.0/I35.1/I35.2/I35.8/I35.9/I36.0/I36.1/I36.2/I36.8/I36.9/I37.0/I37.1/I37.2/I37.8/I37.9/I38/I39.0/I39.1/I39.2/I39.3/I39.4/I39.8/Q23.0/Q23.1/Q23.2/Q23.3/Z95.2/Z95.4	1 year before the cohort entry date
Cerebrovascular disease	At least one of the following diagnoses (ICD-10 codes): G45.0/G45.1/G45.2/G45.3/G45.4/G45.8/G45.9/G46.0/G46.1/G46.2/G46.3/G46.4/G46.5/G46.6/G46.7/G46.8/H34.0/I60.0/I60.1/I60.2/I60.3/I60.4/I60.5/I60.6/I60.7/I60.8/I60.9/I61.0/I61.1/I61.2/I61.3/I61.4/I61.5/I61.6/I61.8/I61.9/I62.0/I62.1/I62.9/I63.0/I63.1/I63.2/I63.3/I63.4/I63.5/I63.6/I63.8/I63.9/I64/I65.0/I65.1/I65.2/I65.3/I65.8/I65.9/I66.0/I66.1/I66.2/I66.3/I66.4/I66.8/I66.9/I67.0/I67.1/I67.2/I67.3/I67.4/I67.5/I67.6/I67.7/I67.8/I67.9/I68.0/I68.1/I68.2/I68.8/I69.0/I69.1/I69.2/I69.3/I69.4/I69.8	1 year before the cohort entry date
Renal disease	At least one of the following diagnoses (ICD-10 codes): I12.0/I13.1/N03.2/N03.3/N03.4/N03.5/N03.6/N03.7/N05.2/N05.3/N05.4/N05.5/N05.6/N05.7/N18.0/N18.8/N18.9/N19/N25.0/Z49.0/Z49.1/Z49.2/Z94.0/Z99.2	1 year before the cohort entry date
Liver disease	At least one of the following diagnoses (ICD-10 codes): B18.0/B18.1/B18.2/B18.8/B18.9/K70.0/K70.1/K70.2/K70.3/K70.9/K71.3/K71.4/K71.5/K71.7/K73.0/K73.1/K73.2/K73.8/K73.9/K74.0/K74.1/K74.2/K74.3/K74.4/K74.5/K74.6/K76.0/K76.2/K76.3/K76.4/K76.8/K76.9/Z94.4<br>I85.0/I85.9/I86.4/I98.2/K70.4/K71.1/K72.1/K72.9/K76.5/K76.6/K76.7	1 year before the cohort entry date
Diabetes	At least one of the following diagnoses (ICD-10 codes): E10.0/E10.1/E10.6/E10.8/E10.9/E11.0/E11.1/E11.6/E11.8/E11.9/E12.0/E12.1/E12.6/E12.8/E12.9/E13.0/E13.1/E13.6/E13.8/E13.9/E14.0/E14.1/E14.6/E14.8/E14.9<br>E10.2/E10.3/E10.4/E10.5/E10.7/E11.2/E11.3/E11.4/E11.5/E11.7/E12.2/E12.3/E12.4/E12.5/E12.7/E13.2/E13.3/E13.4/E13.5/E13.7/E14.2/E14.3/E14.4/E14.5/E14.7	1 year before the cohort entry date
Dementia	At least one of the following diagnoses (ICD-10 codes): F00.0/F00.1/F00.2/F00.9/F01.0/F01.1/F01.2/F01.3/F01.8/F01.	1 year before the cohort entry date

	9/F02.0/F02.1/F02.2/F02.3/F02.4/F02.8/F03/F05.1/G30.0/G30.1/G30.8/G30.9/G31.1	
Rheumatic disease	At least one of the following diagnoses (ICD-10 codes): M05.0/M05.1/M05.2/M05.3/M05.8/M05.9/M06.0/M06.1/M06.2/M06.3/M06.4/M06.8/M06.9/M31.5/M32.0/M32.1/M32.8/M32.9/M33.0/M33.1/M33.2/M33.9/M34.0/M34.1/M34.2/M34.8/M34.9/M35.1/M35.3/M36.0	1 year before the cohort entry date
Hypertension	More than two times in another month during the period<br>At least one of the following diagnoses (ICD-10 codes): I10-I15 with anti-hypertensive medication	1 year before the cohort entry date
Dyslipidemia	More than two times in another month during the period:<br>At least one of the following diagnoses (ICD-10 codes): E78 with anti-hypertensive medication	1 year before the cohort entry date
Number of previous COVID-19 testing	The number of days of the following procedure codes from the claims data: 160175550/160223350/160223450/160223550/160224250/160224750/160224850/160226450/160229550/160229650/160229750/160229850/160229950/160230050	1 year before the cohort entry date

COVID-19, coronavirus disease.

eTable 2. Baseline characteristics in the matched cohort stratified by municipality

	A		B		C		D	
	Vaccinated, N=44,207	Unvaccinated, N=43,440	Vaccinated, N =34,920	Unvaccinated, N=47,502	Vaccinated, N=52,134	Unvaccinated, N=51,962	Vaccinated, N=72,313	Unvaccinated, N=60,670
Age, years, mean (SD)	78.2 (7.3)	78.3 (7.3)	78.3 (7.8)	77.6 (7.5)	78.1 (6.6)	76.1 (6.9)	78.3 (7.4)	77.6 (7.2)
Age groups, years, n (%)								
65–74	15,747 (35.6)	15,323 (35.3)	12,947 (37.1)	18,850 (39.7)	16,685 (32.0)	25,472 (49.0)	25,469 (35.2)	23,671 (39.0)
75–84	19,293 (43.6)	18,867 (43.4)	13,989 (40.1)	19,429 (40.9)	26,582 (51.0)	19,697 (37.9)	31,520 (43.6)	25,884 (42.7)
85–94	8,386 (19.0)	8,418 (19.4)	7,116 (20.4)	8,456 (17.8)	8,238 (15.8)	6,250 (12.0)	14,033 (19.4)	10,306 (17.0)
≥95	781 (1.8)	832 (1.9)	868 (2.5)	767 (1.6)	629 (1.2)	543 (1.0)	1,291 (1.8)	809 (1.3)
Sex, n (%)								
Men	17,674 (40.0)	17,281 (39.8)	14,316 (41.0)	20,030 (42.2)	22,287 (42.7)	22,644 (43.6)	29,244 (40.4)	25,370 (41.8)
Women	26,533 (60.0)	26,159 (60.2)	20,604 (59.0)	27,472 (57.8)	29,847 (57.3)	29,318 (56.4)	43,069 (59.6)	35,300 (58.2)
Cancer, n (%)	8,185 (18.5)	7,990 (18.4)	4,856 (13.9)	6,955 (14.6)	7,595 (14.6)	7,344 (14.1)	11,101 (15.4)	9,682 (16.0)
Chronical pulmonary disease, n (%)	9,589 (21.7)	9,380 (21.6)	5,910 (16.9)	7,942 (16.7)	9,064 (17.4)	8,255 (15.9)	15,782 (21.8)	12,865 (21.2)
Cardiovascular disease, n (%)	17,110 (38.7)	16,885 (38.9)	10,051 (28.8)	13,370 (28.1)	15,204 (29.2)	13,870 (26.7)	26,110 (36.1)	20,892 (34.4)
Cerebrovascular disease, n (%)	10,698 (24.2)	10,530 (24.2)	7,638 (21.9)	9,722 (20.5)	11,181 (21.4)	9,744 (18.8)	13,668 (18.9)	11,008 (18.1)
Renal disease, n (%)	3,561 (8.1)	3,370 (7.8)	1,845 (5.3)	2,415 (5.1)	2,671 (5.1)	2,512 (4.8)	4,097 (5.7)	3,422 (5.6)
Liver disease, n (%)	14,336 (32.4)	13,922 (32.0)	7,027 (20.1)	9,805 (20.6)	8,591 (16.5)	8,179 (15.7)	13,461 (18.6)	11,275 (18.6)
Diabetes, n (%)	4,784 (10.8)	4,736 (10.9)	3,547 (10.2)	4,933 (10.4)	4,484 (8.6)	4,473 (8.6)	7,964 (11.0)	6,921 (11.4)
Dementia, n (%)	4,022 (9.1)	3,920 (9.0)	3,210 (9.2)	2,971 (6.3)	3,965 (7.6)	3,283 (6.3)	7,282 (10.1)	4,804 (7.9)
Rheumatic disease, n (%)	2,217 (5.0)	2,120 (4.9)	1,143 (3.3)	1,642 (3.5)	2,187 (4.2)	2,127 (4.1)	3,417 (4.7)	2,923 (4.8)
Hypertension, n (%)	26,517 (60.0)	25,958 (59.8)	20,960 (60.0)	28,132 (59.2)	30,115 (57.8)	27,899 (53.7)	43,490 (60.1)	35,483 (58.5)
Dyslipidemia, n (%)	19,207 (43.4)	18,417 (42.4)	13,368 (38.3)	18,177 (38.3)	19,540 (37.5)	18,356 (35.3)	30,413 (42.1)	24,663 (40.7)
Number of clinic/hospital visits in the past year, Median (IQR)	29.5 (34.1)	28.0 (32.0)	22.5 (25.7)	21.5 (24.7)	22.6 (26.7)	20.4 (25.6)	28.0 (29.0)	26.1 (27.7)
Number of previous COVID-19 testing, n (%)								
0	43,052 (97.4)	42,202 (97.2)	31,747 (90.9)	43,107 (90.7)	48,158 (92.4)	47,678 (91.8)	67,933 (93.9)	56,636 (93.4)
1	1,020 (2.3)	1,072 (2.5)	2,369 (6.8)	3,152 (6.6)	3,056 (5.9)	3,226 (6.2)	3,602 (5.0)	3,250 (5.4)
≥2	135 (0.3)	166 (0.4)	804 (2.3)	1,243 (2.6)	920 (1.8)	1,058 (2.0)	778 (1.1)	784 (1.3)

COVID-19, coronavirus disease; IQR, interquartile range; SD, standard deviation.

eTable 3. Effectiveness of BNT162b2 vaccine in the older adults aged ≥65 years during the Delta-predominant period (August 1, 2021–September 30, 2021)

	Number at risk at	Events	Events/10,000	Risk difference	Vaccine effectiveness

	the start of follow-up		persons (95% CI) [a]	(95% CI) [a, b]	(95% CI) [a]
**Infection**					
Unvaccinated	11,056	12	12.6 (5.7–21.5)	Ref	Ref
0–13 days after first dose	11,056	12	9.1 (4.2–14.7)	3.5 (-5.3 to 13.4)	32.3 (-80.7 to 73.8)
Unvaccinated	11,237	2	2.9 (0.0–7.2)	Ref	Ref
14–20 days after first dose or the day before second dose	11,237	9	7.0 (3.0–12.2)	-4.1 (-10.4 to 1.6)	-179.3 (-775.3 to 21.0)
13 days after the second dose					
Unvaccinated	37,477	10	3.4 (1.4–5.6)	Ref	Ref
21–27 days after first dose or 0-6 days after the second dose	37,477	11	4.1 (1.7–7.0)	-0.7 (-4.2 to 2.7)	-7.4 (-190.0 to 62.2)
Unvaccinated	22,919	74	32.7 (25.3–40.7)	Ref	Ref
7 days or more after the second dose	22,919	18	7.6 (4.3–11.3)	25.1 (17.0–33.8)	78.0 (63.5–87.8)
**Symptomatic infection**					
Unvaccinated	11,056	11	11.3 (4.8–20.0)	Ref	Ref
0–13 days after first dose	11,056	12	9.1 (4.2–14.7)	2.2 (-6.5 to 11.9)	24.3 (-104.8 to 71.2)
Unvaccinated	11,238	2	2.9 (0.0–7.6)	Ref	Ref
14–20 days after first dose or the day before second dose	11,238	6	4.7 (1.5–8.8)	-1.8 (-7.4 to 3.9)	-89.5 (-542.5 to 63.1)
13 days after the second dose					
Unvaccinated	13,998	5	5.2 (1.0–10.3)	Ref	Ref
21–27 days after first dose or 0-6 days after the second dose	13,998	7	5.9 (1.8–11.0)	-0.7 (-7.5 to 5.9)	0.5 (-359.1 to 73.8)
Unvaccinated	22,927	64	28.5 (21.9–35.7)	Ref	Ref
7 days or more after the second dose	22,927	14	6.0 (2.9–9.4)	22.6 (15.1–29.8)	80.3 (66.8–90.6)
**Hospitalization**					
Unvaccinated	11,062	2	1.4 (0.0–3.7)	Ref	Ref
0–13 days after first dose	11,062	0	-	-	-
Unvaccinated	11,267	2	2.1 (0.0–5.5)	Ref	Ref
14–20 days after first dose or the day before second dose	11,267	1	0.7 (0.0–2.0)	1.4 (-1.3 to 4.9)	64.2 (-93.2 to 100.0)
13 days after the second dose					
Unvaccinated	14,044	2	1.8 (0.0–4.7)	Ref	Ref
21–27 days after first dose or 0-6 days after the second dose	14,044	2	1.5 (0.0–3.7)	0.3 (-2.8 to 3.9)	23.9 (-289.9 to 100.0)
Unvaccinated	22,997	43	18.2 (12.5–23.9)	Ref	Ref
7 days or more after the second dose	22,997	2	0.8 (0.0–2.1)	17.4 (11.5–22.8)	94.7 (84.8–100.0)

CI, confidence interval.

[a] Adjusted by inverse probability weighting using the propensity score.

[b] The risk differences were calculated as unvaccinated minus vaccinated groups.

eTable 4. BNT162b2 vaccine effectiveness using Cox proportional hazard model during the Delta-predominant period (July 1, 2021-September 30, 2021)

	Number at risk at the start of follow-up	Events	Unadjusted HR (95% CI)	Adjusted HR [a] (95% CI)	Vaccine effectiveness[b] (95% CI)
**Infection**					
Unvaccinated	58,185	26	Ref.	Ref.	Ref.
0–13 days after the first dose	58,185	25	0.962 (0.555–1.665)	1.052 (0.606–1.827)	3.8 (−66.5 to 44.5)
Unvaccinated	42,632	16	Ref.	Ref.	Ref.
14–20 days after the first dose or the day before the second dose or 14 days after the second dose	42,632	12	0.75 (0.355–1.585)	0.96 (0.451–2.043)	25 (−58.5 to 64.5)
Unvaccinated	37,477	10	Ref.	Ref.	Ref.
21–27 days after the first dose or 0–6 days after the second dose	37,477	11	1.099 (0.467–2.588)	1.373 (0.581–3.247)	−9.9 (−158.8 to 53.3)
Unvaccinated	34,039	90	Ref.	Ref.	Ref.
7 days after the second dose	34,039	22	0.24 (0.151–0.383)	0.266 (0.166–0.425)	76 (61.7–84.9)
**Symptomatic infection**					
Unvaccinated	58,185	23	Ref.	Ref.	Ref.
0–13 days after the first dose	58,185	23	1 (0.561–1.782)	1.1 (0.616–1.967)	−10 (−96.7 to 38.4)
Unvaccinated	42,635	14	Ref.	Ref.	Ref.
14–20 days after the first dose or the day before the second dose or 14 days after the second dose	42,635	9	0.643 (0.278–1.485)	0.777 (0.335–1.802)	22.3 (−80.2 to 66.5)
Unvaccinated	37,485	8	Ref.	Ref.	Ref.
21–27 days after the first dose or 0–6 days after the second dose	37,485	9	1.124 (0.434–2.914)	1.412 (0.542–3.68)	−41.2 (−268 to 45.8)
Unvaccinated	34,050	78	Ref.	Ref.	Ref.
7 days after the second dose	34,050	18	0.227 (0.136–0.378)	0.252 (0.15–0.422)	74.8 (57.8–85)
**Hospitalization**					
Unvaccinated	58,186	4	Ref.	Ref.	Ref.
0–13 days after the first dose	58,186	1	0.197 (0.023–1.701)	0.2 (0.023–1.712)	80 (−71.2 to 97.7)
Unvaccinated	42,670	2	Ref.	Ref.	Ref.
14–20 days after the first dose or the day before the second dose or 14 days after the second dose	42,670	2	1.031 (0.144–7.357)	1 (0.141–7.099)	0 (−609.9 to 85.9)
Unvaccinated	37,540	3	Ref.	Ref.	Ref.
21–27 days after the first dose or 0–6 days after the second dose	37,540	2	0.581 (0.096–3.509)	0.666 (0.111–3.987)	33.4 (−298.7 to 88.9)
Unvaccinated	34,110	46	Ref.	Ref.	Ref.

7 days after the second dose	34,110	3	0.055 (0.017–0.177)	0.064 (0.02–0.205)	93.6 (79.5–98)

CI, confidence interval; HR, hazard ratio.
[a] Adjusted by inverse probability weighting using the propensity score.
[b] Vaccine effectiveness was calculated as 1 minus adjusted HR.

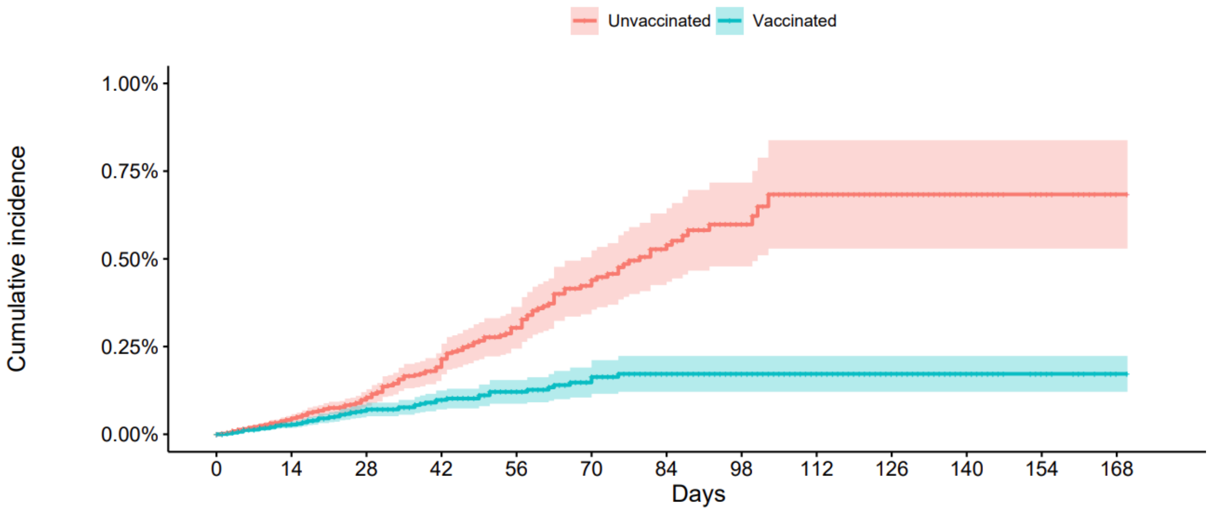

Number at risk

	0	14	28	42	56	70	84	98	112	126	140	154	168
Unvaccinated	203574	86194	42666	25528	17494	12090	8197	4207	1435	533	119	27	4
Vaccinated	203574	86214	42871	26017	18039	12667	8625	4341	1560	605	127	34	4

Cumulative number of events

	0	14	28	42	56	70	84	98	112	126	140	154	168
Unvaccinated	0	58	93	128	147	167	177	181	184	184	184	184	184
Vaccinated	0	36	62	70	75	81	82	82	82	82	82	82	82

eFigure 1. Cumulative incidence of SARS-CoV-2 infection in the matched cohort between April 12, 2021 and September 30, 2021

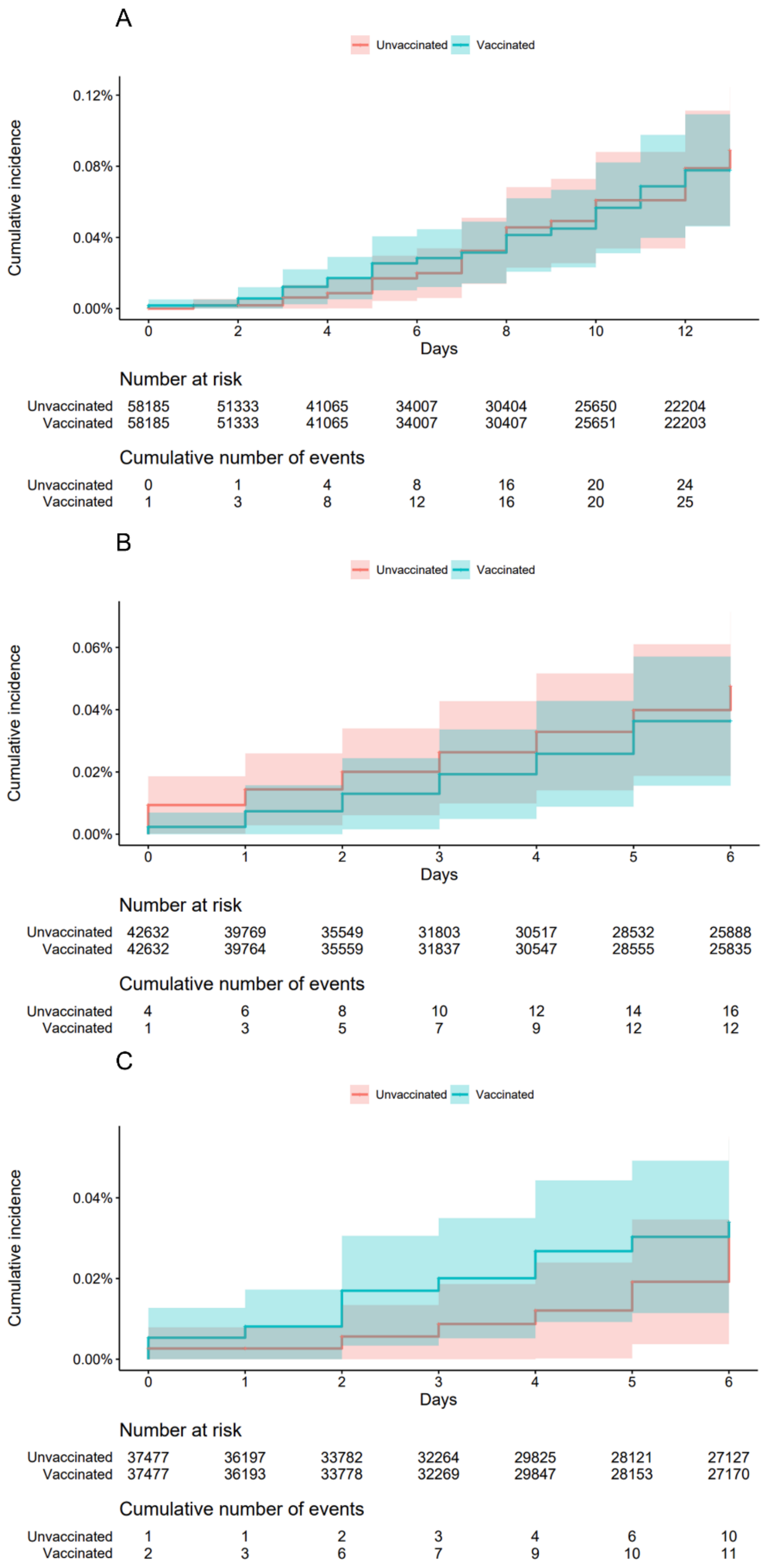

eFigure 2. Cumulative incidence of SARS-CoV-2 infection after restricting the population still at risk at the start each period. (**A**) 0–13 days after the first dose, (**B**) 14–20 days after the first dose or the day before the second dose or 14 days after the second dose, (**C**) 21–27 days after the first dose or 0–6 days after the second dose

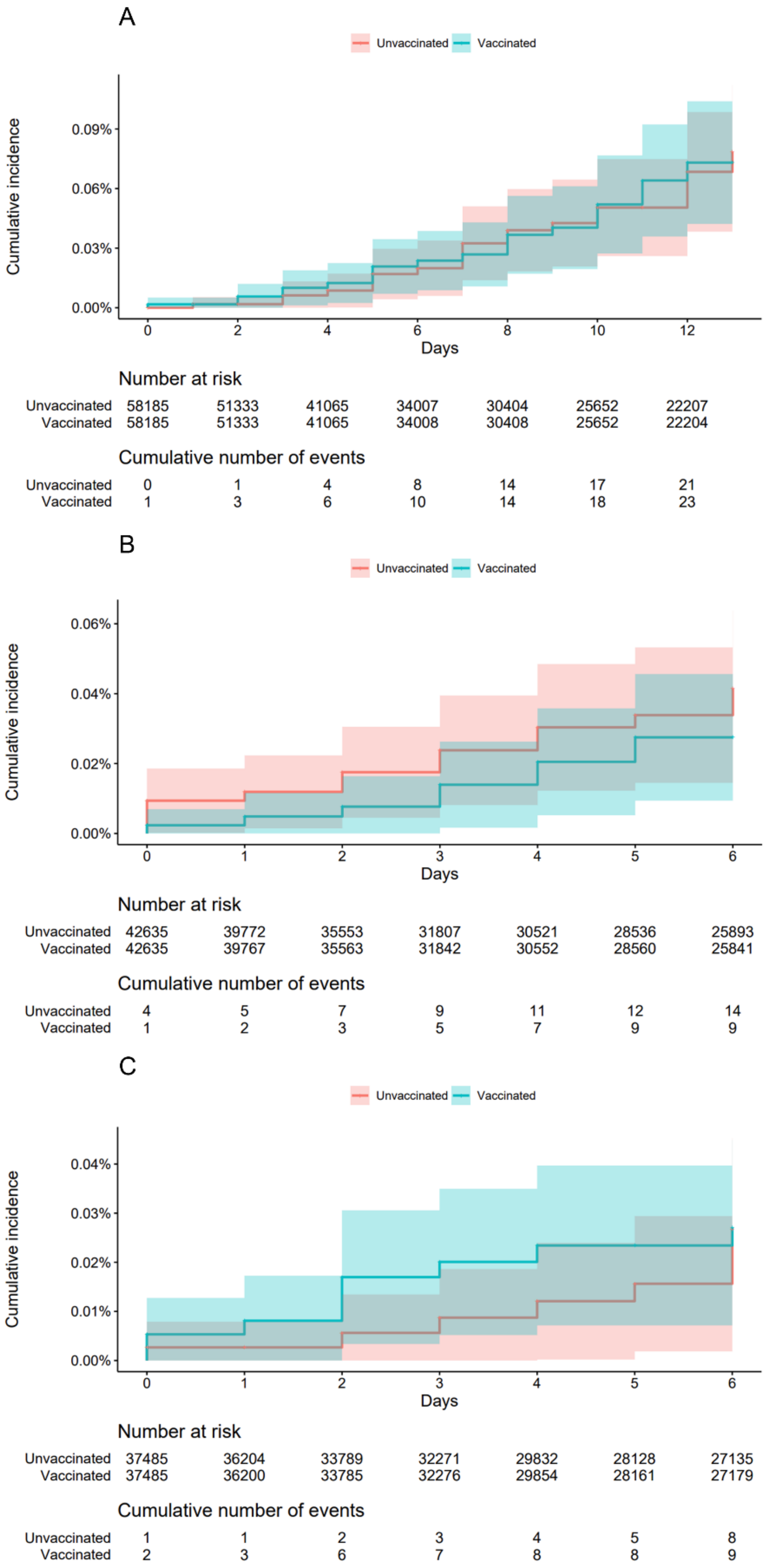

eFigure 3. Cumulative incidence of symptomatic SARS-CoV-2 infection after restricting the population still at risk at the start each period. (**A**) 0–13 days after the first dose, (**B**) 14–20 days after the first dose or the day before the second dose or 14 days after the second dose, (**C**) 21–27 days after the first dose or 0–6 days after the second dose

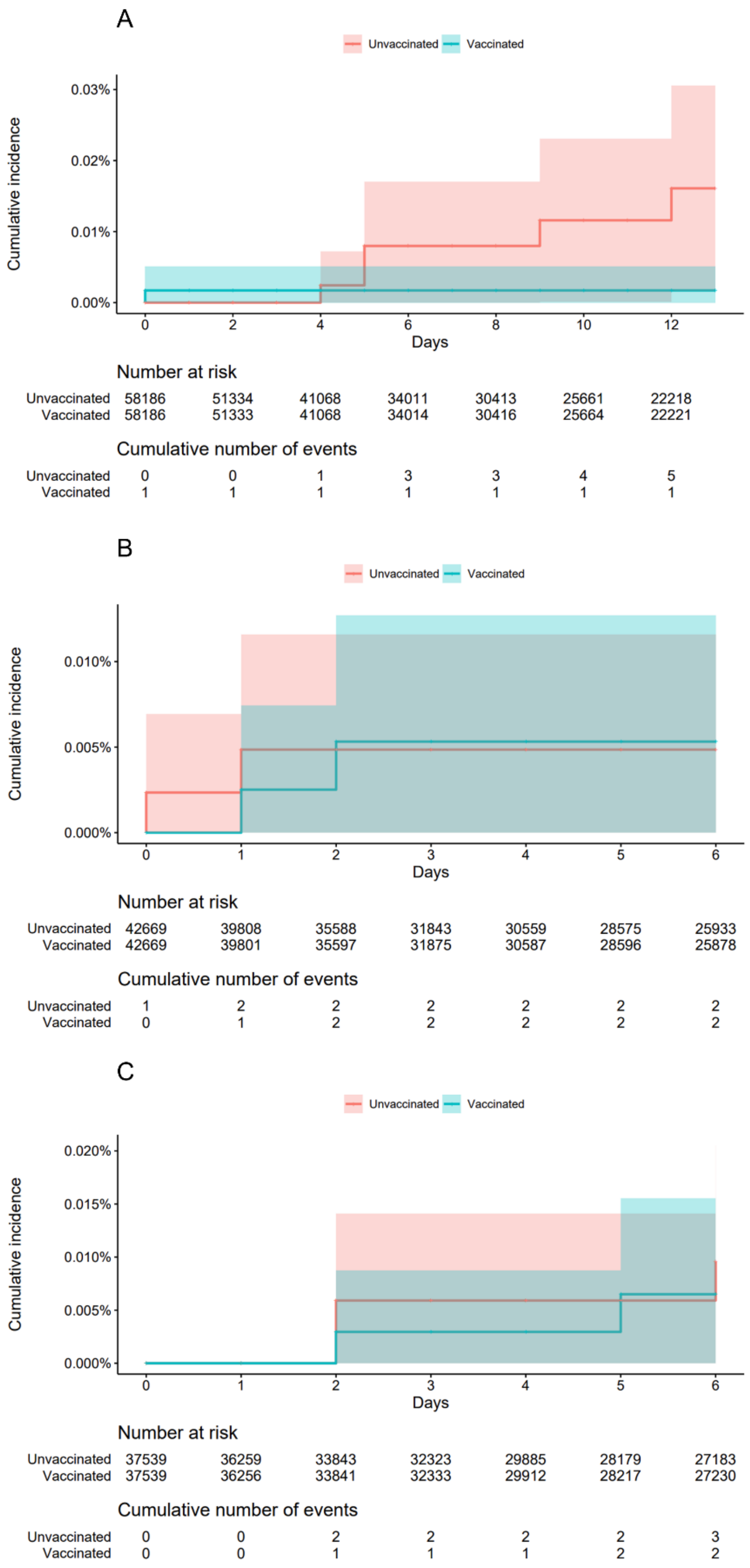

eFigure 4. Cumulative incidence of hospitalization related to SARS-CoV-2 infection in the population still at risk at the start each period. (**A**) 0–13 days after the first dose, (**B**) 14–20 days after the first dose or the day before the second dose or 14 days after the second dose, (**C**) 21–27 days after the first dose or 0-6 days after the second dose
